# Supplementary material for: Motor control exercises versus general exercises for greater trochanteric pain syndrome: A protocol of a randomized controlled trial
Source: PLoS One. 2022 Jun 24;17(6):e0269230. doi: 10.1371/journal.pone.0269230 (PMC9231741; doi:10.1371/journal.pone.0269230)
Supplement: S1 Protocol — (DOCX) [file pone.0269230.s002.docx]

**INTRODUÇÃO**

A síndrome dolorosa do trocânter maior (SDTM) é um termo abrangente usado para definir a dor e a sensibilidade na região do trocânter maior do fêmur^1,2^. Estima-se que 10 a 25% da população desenvolverá algum tipo de dor na região lateral do quadril^1–3^, predominantemente em mulheres com mais de 40 anos^2–6^. A SDTM impacta diretamente na piora da qualidade de vida, o que é semelhante aos achados em indivíduos com osteoartrite de quadril grave^7^. As mulheres apresentam mecanismos que podem influenciar no aparecimento da SDTM, como menores inserções tendíneas glúteas no fêmur, braço do momento glúteo mais curto e fraqueza do glúteo médio^6^.

Mudanças na função dos músculos glúteo médio e mínimo podem levar ao mau controle do quadril e pélvico e levar ao aumento da adução do quadril no plano frontal, principalmente em posições de apoio unilateral, como na marcha^6,8^. A literatura indica que os tratamentos ativos, aqueles em que os participantes realizam exercícios físicos, são preferíveis às abordagens passivas no tratamento da SDTM. Os tratamentos ativos resultam em melhores resultados em curto^9,10^, médio^9^ e longo prazo^9-11^ em termos de dor e função quando comparados ao repouso, terapia por ondas de choque e / ou tratamentos invasivos, por exemplo, injeção de corticosteroide^5,9,11^. As intervenções cirúrgicas geralmente são reservadas para os casos recalcitantes^1,2,5,12^.

Apesar do conhecimento prévio de que o exercício físico gera benefícios para a SDTM e a dor crônica em geral, nenhum estudo tentou demonstrar a eficácia do exercício de controle motor para a SDTM. Indivíduos com SDTM têm controle anormal dos movimentos dos membros inferiores e parâmetros neuromusculares deficientes^8,13,14^ e alterações no tronco e cinemática pélvica durante a caminhada^15^, no entanto, nenhum estudo utilizou o treinamento neuromuscular como estratégia de tratamento e não há evidências suficientes sobre a influência deste intervenção nos aspectos clínicos e biomecânicos desses participantes. Assim, este estudo tem como objetivo comparar o efeito de um protocolo de exercícios gerais versus um programa de treinamento de controle motor sobre a dor no início e após o tratamento em 8 e 60 semanas em mulheres com SDTM.

Nossa hipótese é que tanto as mulheres submetidas ao protocolo de controle motor quanto as mulheres submetidas ao protocolo geral de exercícios apresentarão melhorias em todos os desfechos avaliados, embora o grupo de controle motor seja superior devido à intervenção ser específica para a região do quadril.

**MÉTODOS**

Este é um ensaio controlado aleatorizado, cego para avaliador e estatístico, com 2 braços, registrado no Registro Brasileiro de Ensaios Clínicos (RBR-37gw2x) e tem um Número Universal de Ensaio (U1111-1234-0705)

**Aprovação e consentimento éticos**

O estudo foi aprovado pelo Comitê de Ética em Pesquisa da Faculdade de Filosofia e Ciências da Universidade Estadual Paulista (UNESP), Campus de Marília (CAAE: 87372318.1.0000.5406). Todos as participantes assinarão um termo de consentimento livre e esclarecido.

**Participantes**

**Recrutamento**

As mulheres com SDTM serão recrutadas na comunidade, universidades e dentro do serviço público de saúde, por meio digital (facebook e instagram) e divulgação escrita (panfletos). As participantes interessadas entrarão em contato com o pesquisador principal por telefone ou redes sociais.

**Triagem clínica**

**Triagem por telefone**

A triagem inicial será feita por telefone. As participantes potenciais serão elegíveis para o estudo se tiverem entre 18 e 70 anos, não praticarem atividade física regular (nível de atividade de Tegner ≤4)^16^ e apresentarem dor lateral no quadril por ≥ 3 meses. Devem apresentar dor durante uma ou mais das sete atividades diárias: caminhar, ficar em pé por um período prolongado, levantar-se da posição sentada, sentar-se por muito tempo, subir e descer escadas, subir e descer ladeiras e deitar-se de lado. As participantes potenciais serão inelegíveis para o estudo se tiverem um índice de massa corporal (IMC)> 36 kg/m2, tiverem recebido algum tipo de intervenção invasiva para dor lateral do quadril ou intervenção de fisioterapia para dor no quadril nos últimos 12 semanas, ter rigidez matinal no quadril ≤ 60 minutos, qualquer doença que afete o sistema neuromuscular ou que possa impedir a coleta de dados, tenha feito cirurgia na coluna ou no quadril, qualquer condição infecciosa, qualquer neoplasia, ou não possa se comprometer a participar durante o tratamento^10-12,15,17^.

**Avaliação fisioterapêutica**

As participantes em potencial que atenderem aos critérios de elegibilidade por telefone serão submetidas a duas avaliações. A primeira será realizada para triagem dos critérios de inclusão e exclusão que não foram possíveis de avaliar por telefone, bem como explicar com mais detalhes a natureza do estudo e o comprometimento exigido, com oportunidade para as participantes fazerem perguntas, e o segundo será realizado por um avaliador cego para avaliar os desfechos. A avaliação fisioterapêutica será realizada na Universidade Estadual Paulista e levará cerca de 40 minutos a 1 hora. A avaliação será feita por uma fisioterapeuta credenciada com 8 anos de experiência, com mestrado na área de distúrbios musculoesqueléticos. Serão coletados dados demográficos, incluindo idade, peso, altura, IMC etc. Será determinado se as participantes têm a capacidade de manipular sapatos e meias^18^, têm limitação na amplitude de movimento da coluna, quadril ou membro inferior que afeta a marcha ou a coleta de dados ou discrepância visível nos membros inferiores. Será avaliado se as participantes apresentam sensibilidade à palpação sobre a região do trocânter maior do fêmur^8,19,20^. Se ambos os quadris forem sintomáticos, o lado mais dolorido será avaliado^10^. O sinal de Trendelenburg, que é a queda pélvica durante o apoio unipodal, indicando ineficiência dos músculos abdutores do quadril, será avaliado^5,8,19^.

Os testes que serão utilizados para avaliação clínica têm como objetivo definir a localização exata da dor e transmitir forças compressivas e / ou de tensão nos tendões dos músculos abdutores do quadril sobre o trocânter maior. As participantes devem sentir dor sobre o trocânter maior do fêmur em ≥ dois dos sete testes clínicos provocativos de dor^12,14,17^. Eles são:

• Palpação do trocanter maior: sensibilidade à palpação positiva, considerada quando há dor sobre o trocanter maior do fêmur, durante a avaliação em decúbito lateral (DL) com o lado da dor para cima, flexão da articulação do quadril e joelhos juntos^5,6, 8,11,17^. Foi relatado que esse teste tem sensibilidade de 85,7% e especificidade de 61,1 %^6^.

• FADER: Participantes em decúbito dorsal (DD), devem relatar dor sobre o trocânter maior quando o membro inferior afetado é posicionado em flexão de 90º, adução e rotação externa do quadril^17^.

• FADER-R com teste muscular estático: Participantes em DD, tem que relatar dor quando o membro inferior afetado é posicionado da mesma forma, mas mantendo resistência isométrica à rotação interna ao final da amplitude de movimento^5,6,8,11,17^. Este teste é uma modificação do teste de rotação externa resistida, que foi relatado como tendo 42,3% de sensibilidade e 95% de especificidade^6^.

• FABER: Participantes em DD, deve relatar dor quando o membro inferior afetado é posicionado em flexão, abdução e rotação externa do quadril^6,17,18^. Foi relatado que esse teste tem sensibilidade de 50% e especificidade de 83 %^18^.

• ADD: As participantes devem relatar dor quando o membro inferior afetado é posicionado em adução passiva do quadril em DL^17^.

• ADD R: As participantes devem relatar dor quando o membro inferior afetado é posicionado em adução passiva do quadril em DL, mas mantendo a resistência isométrica em abdução^17^. Foi relatado que esse teste tem sensibilidade de 50% e especificidade de 97,3 %^6^.

• Posicionamento unipodal: As participantes devem relatar dor ao se apoiar em uma perna por 30 segundos. Este teste tem sensibilidade de 45,4% e especificidade de 84,2% para indicar SDTM^6^.

**Tamanho da amostra e análise do poder**

O cálculo amostral foi realizado por meio do software G * Power, com base na medida do nível de dor, por ser o desfecho primário da pesquisa. Utilizou-se um poder de 0,80, probabilidade de erro α 0,05, tamanho de efeito de 0,5 e taxa de abandono de 15%, para isso recrutaremos 60 participantes (30 em cada grupo)^9^.

**Randomização, alocação e cegamento**

Se todos os critérios de elegibilidade forem atendidos, e se a presença da SDTM for determinada, as participantes serão aleatoriamente designadas para receber 1) exercícios de controle motor ou 2) exercícios gerais. As participantes serão alocados aleatoriamente em dois braços terapêuticos usando randomização em bloco permutada para equilibrar o número de pacientes alocadas para cada grupo. A sequência de randomização do bloco permutado (com seis pacientes por bloco) será gerada pelo site www.sealedenvelope.com. As participantes serão informadas de sua alocação aleatória por um dos pesquisadores não envolvidos no processo de avaliação. Após a randomização, as participantes serão convidadas a retornar para avaliação fisioterapêutica, com uma avaliadora cega, fisioterapeuta credenciada com 8 anos de experiência e doutorado na área de distúrbios musculoesqueléticos. Esta avaliadora não participará da triagem ou intervenções. Todas as participantes serão aconselhadas a não divulgar ao avaliador quaisquer detalhes sobre o programa de intervenção que tenham recebido (para garantir que a alocação seja oculta). Nesta avaliação, as participantes darão seu consentimento livre e esclarecido.

**Avaliação dos desfechos**

**Desfecho primário**

Existe uma medida de desfecho primário: 1) Dor média na semana anterior.

1) A intensidade da dor será avaliada no início e após o tratamento em 8 semanas. Ela também será avaliado em 60 semanas, que é um ponto de tempo secundário para o resultado primário. A intensidade da dor será avaliada pela escala visual analógica (EVA)^5^. Formada por uma linha horizontal de 100 mm, ancorada pelas palavras “sem dor / desconforto” e “pior dor / desconforto imaginável”^5^. Para medir a dor, a participante será solicitada a indicar o nível de dor que está sentindo no momento e a dor média que sentiu na última semana, marcando a escala com uma linha. Uma régua será usada para medir o valor obtido^5^.

**Desfechos secundários**

Existem sete medidas de resultados secundários: 1) Efeito global percebido, 2) Força muscular, 3) Catastrofização da dor, 4) Cinesiofobia, 5) Sensibilização central, 6) Padrões de recrutamento muscular, 7) Funcionalidade do quadril

1) A escala de efeito percebido global (EGP) será utilizada após o tratamento em 8 e 60 semanas^21^. Esta é uma escala de 7 pontos (1 = completamente recuperado, 7 = pior do que nunca) para avaliar a recuperação. EGP avalia a percepção da participante em relação à modificação de sua condição clínica após a intervenção, e é avaliada por uma pergunta simples e de fácil compreensão com opções de resposta alternativas que serão dicotomizadas em "melhorado" ("completamente recuperado" e "muito melhor") versus “Não melhorou” (“melhorou ligeiramente”, “não mudou”, “piorou ligeiramente”, “piorou muito”, “pior do que nunca”^21^.

2) A força isométrica dos músculos abdutores e extensores do quadril será avaliada no início e após o tratamento, 8 semanas durante a reavaliação pós-intervenção. Será utilizado um dinamômetro manual modelo Lafayette (Lafayette Instruments), que tem se mostrado um método válido de avaliação da força de contração muscular isolada^22^. Para avaliação da musculatura abdutora do quadril, as participantes serão posicionadas em decúbito dorsal sobre uma maca, estabilizada por faixas de velcro ao redor da pelve e acima do maléolo lateral do tornozelo para evitar movimentos compensatórios e a influência da resistência do examinador^22^. O dinamômetro será posicionado acima do maléolo lateral do tornozelo e fixado à maca por uma faixa inelástica, será posicionado sem rotação, com 10º de abdução do quadril para minimizar o potencial de compressão dos tendões contra o trocanter maior^14^. O membro inferior não testado será posicionado com 45º de flexão de quadril e joelho, com o pé posicionado na maca e os membros superiores apoiados ao lado do corpo (figura 2)^14^. Para avaliação dos extensores do quadril, as participantes serão posicionadas na posição prona com flexão do joelho no membro inferior a ser avaliado. O dinamômetro será posicionado na parte posterior da coxa, acima da fossa poplítea e será estabilizado por uma faixa de velcro inelástica^23^. Outra faixa de velcro será posicionada ao redor da pelve (figura 3)^23^. A mensuração do comprimento dos membros será realizada com fita métrica de tecido padrão para o cálculo do torque, desde o trocânter maior até o centro do dinamômetro, e será registrada em metros^23,24^. Os testes de força isométrica máxima serão realizados 3 vezes com 5 segundos de duração para cada contração, com 30 segundos de descanso entre cada tentativa^23^. As participantes serão encorajadas verbalmente a executar o máximo de força possível durante o teste. Os valores de força serão normalizados pelo peso de cada participante^23^.

Figura 2. Avaliação da força isométrica dos músculos abdutores do quadril


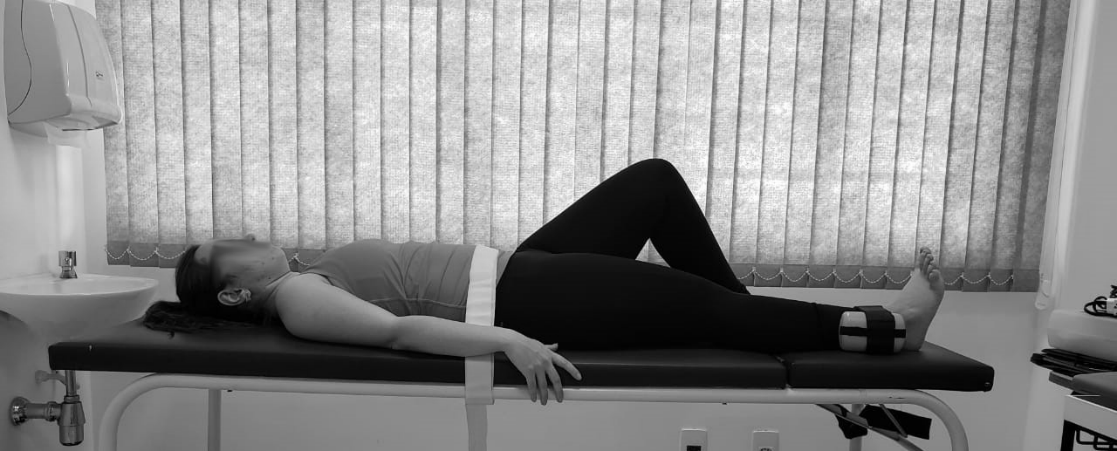


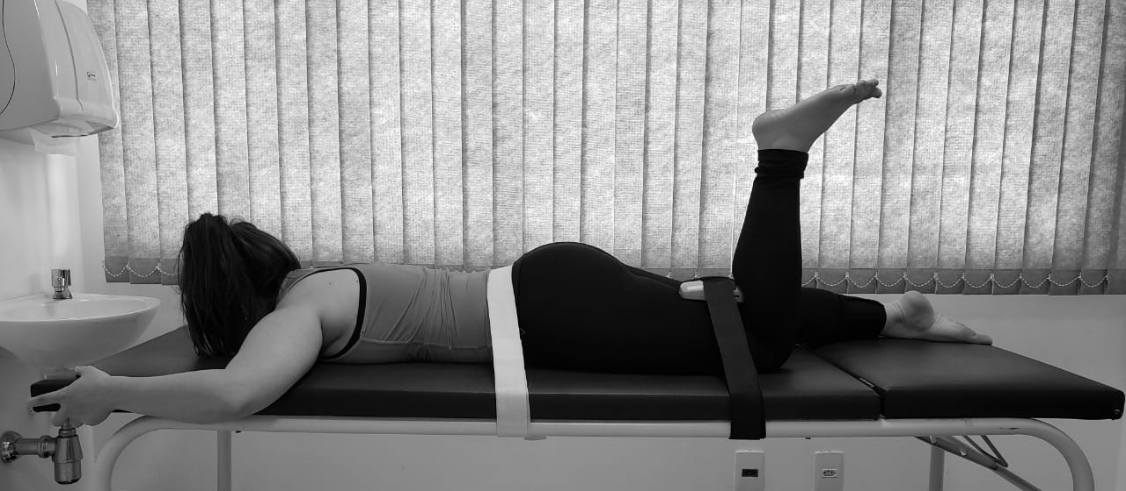
Figura 3. Avaliação da força isométrica dos músculos extensores do quadril

3) A catastrofização da dor será medida com a Escala de Catastrofização da Dor (ECD) no início e após o tratamento, em 8 e 60 semanas durante a reavaliação pós-intervenção. A ECD é um questionário validado^25^, autoaplicável, de 13 itens que avalia pensamentos, sentimentos e comportamento catastróficos quando com dor^25^. Este questionário avalia três domínios principais: desamparo, magnificação e ruminação em relação à dor. Os resultados são calculados pela soma de todos os itens da pesquisa e os escores totais variam de 0 a 52, com escores mais altos indicando níveis mais elevados de catastrofização da dor^25^.

4) A cinesiofobia será avaliada com a escala Tampa de cinesiofobia, que é um questionário validado, autoaplicável, com 17 questões e traduzido para o português. A cinesiofobia será avaliada no início e após o tratamento, às 8 e 60 semanas durante a reavaliação pós-intervenção. O escore final pode ser de no mínimo 17 e no máximo 68 pontos, sendo que escores mais altos indicam graus mais elevados de cinesiofobia^26^.

5) A sensibilização central será medida com o inventário de sensibilização central, que é um questionário de sintomas de saúde autoaplicável, projetado como um rastreador de fácil administração para pacientes com alto risco de sensibilização central ou para avaliação de sintomas relacionados à sensibilização central^27^. A sensibilização central será medida no início e após o tratamento em 8 e 60 semanas durante a reavaliação pós-intervenção. Também tem sido recomendado como um componente de um algoritmo para ajudar a classificar pacientes com dor crônica com sensibilização central e para ajudar a diferenciá-los de pacientes com dor neuropática e nociceptiva primária^27^.

6) Os padrões de recrutamento muscular durante a marcha em esteira (MOVEMENT®) serão avaliados no início e após o tratamento em 8 semanas durante a reavaliação pós-intervenção. A eletromiografia de superfície será usada em três diferentes condições de marcha: velocidade preferencial, velocidade máxima mas sem corrida e 3,0km / h. Cada ciclo de velocidade será registrado por um minuto de duração. A familiarização com a esteira será realizada por 5 minutos na velocidade auto-selecionada, o intervalo entre cada teste de cada velocidade será de 1 minuto e os participantes farão o teste descalço^13^. Os sinais eletromiográficos serão captados utilizando um módulo de 8 canais de modelo de aquisição de sinais biológicos (New Miotool Wireless, Porto Alegre, Brasil) com ganho de 2000, conversor A / D de 16 bits, impedância de entrada de 1010 Ohm / 2 pF, CMRR a 126 dB comum, filtro de passagem de banda de 20–500 Hz e taxa de amostragem de 2 kHz. Além disso, o software permite a filmagem em tempo real de cada uma das três velocidades de marcha por meio da webcam. Todos os fios serão unidos e fixados para evitar artefato de movimento. Os músculos de cada participante serão localizados, de acordo com o grupo SENIAM, e os eletrodos serão posicionados unilateralmente no lado afetado (ou mais dolorido se bilateral) e orientados longitudinalmente com as fibras musculares do músculo a ser avaliado^10,13^. A tricotomia será realizada na área de colocação do eletrodo e abrasão da pele com gaze para redução da impedância, além do uso de álcool para limpeza da pele^13,14,15^. Serão utilizados eletrodos bipolares ativos, os quais serão colocados em configuração bipolar, com área de captura de 1cm de diâmetro e distância de 2cm entre eles^13,14^. Um eletrodo de referência será posicionado sobre o processo estilóide da ulna. Os músculos avaliados são:

• Glúteo médio (GMED): os eletrodos serão colocados a 50% ao longo de uma linha desde a crista ilíaca até o ponto mais lateral do trocanter maior.

• Glúteo máximo (GMAX): Os eletrodos serão colocados a 50% ao longo de uma linha entre as vértebras sacrais e o ponto mais lateral do trocanter maior. Essa posição corresponde à maior proeminência do meio das nádegas, bem acima da saliência visível do trocanter maior.

Tensor da fáscia lata (TFL): Os eletrodos serão colocados proximalmente a 1/6 do caminho ao longo de uma linha que vai da espinha ilíaca ântero-superior (EIA) até o côndilo femoral lateral.

7) A funcionalidade do quadril será medida usando o questionário do Victorian Institute of Sport Assessment para tendinopatia glútea (VISA-G) e será avaliada no início e após o tratamento, 8 e 60 semanas durante a reavaliação pós-intervenção. Este é um questionário autoaplicável, traduzido, adaptado transculturalmente e validado para o português brasileiro^33^. Visa-G é específico para avaliar a gravidade da deficiência em pessoas com tendinopatia glútea. É um questionário de 8 itens que avalia dor e função. Os escores variam de 0 a 100 e valores mais altos indicam menos dor e melhor funcionalidade^33^.

7) A incapacidade relacionada ao quadril será medida com o Hip Outcome Score (HOS), que é um questionário autoaplicável para avaliar pacientes jovens, fisicamente ativos ou ambos com distúrbios do quadril. É composto por 28 itens divididos em duas subescalas: atividades da vida diária e atividades esportivas. A pontuação total de cada subescala varia de 0 a 100, onde pontuações mais altas denotam melhor função do quadril. Os escores de cada subescala foram calculados separadamente^34^.

7) A incapacidade também será avaliada por meio do International Hip Outcome Tool (iHOT) que é um questionário autoaplicável de 12 questões. As questões são avaliadas por meio da escala visual analógica, sendo assim, cada questão apresenta uma linha de 10 centímetros e as participantes devem adicionar uma linha vertical cruzando a linha horizontal, sendo que quanto mais à esquerda, pior os sintomas. O resultado de cada questão pode variar entre 0 e 100 e para determinar o resultado deve-se somar todas as questões e dividir pelo número de questões respondidas^35^.

**Análise dos dados eletromiográficos**

A análise eletromiográfica será realizada no software Matlab®. O filtro passa-alto Butterworth de quarta ordem com frequência de corte de 20 Hz, o filtro passa-baixo Butterworth de quarta ordem com frequência de corte de 500 Hz, retificação de sinal de onda completa e filtro passa-baixo de 6 Hz formarão o envelope linear. Os valores do envelope linear dos músculos serão normalizados pelo maior valor obtido na contração isométrica voluntária máxima (CIVM), na avaliação e na reavaliação, de seus respectivos músculos.

**Intervenções**

**Grupo controle motor (GCM)**

O programa de controle motor será realizado ao longo de 8 semanas, com duas consultas semanais, presenciais e individualizadas. Haverá um total de 16 atendimentos, cada um com duração de 50 a 60 minutos. O protocolo consistirá em exercícios de fortalecimento isotônico e isométrico, com foco nos músculos abdutores e extensores do quadril com coordenação por meio de comandos verbais para melhorar o controle motor dinâmico dos membros inferiores. A progressão dos exercícios ocorrerá por meio de faixas elásticas, dos mais fáceis aos mais difíceis, e com adição de exercícios mais difíceis^23^. Na primeira semana não haverá uso de carga, porém a partir da segunda semana o GCM será testado com três cores diferentes de faixas elásticas, da mais fácil à mais difícil e serão instruídas a realizar 3-5 repetições com cada faixa^23^.

Elas vão decidir qual faixa elástica sentem que seriam capazes de realizar 3 séries com 8-12 repetições, mantendo a qualidade do movimento^23^. As participantes escolherão a cor do elástico para cada exercício e o elástico será posicionado acima da articulação do joelho. O teste de progressão da carga será realizado semanalmente e a progressão da carga será aumentada de acordo com a escala de Borg modificada (0-10), quando 3 (moderado) ou escores menores (fácil) forem alcançados, a progressão da carga será feita para os subsequentes elásticos^23^. O descanso entre os exercícios será de um minuto^28^.

Quanto à evolução dos exercícios, caso as participantes não consigam progredir por dores suficientes para desistir do exercício, ou dificuldade em realizar pelo menos 8 repetições com qualidade, elas permanecerão com os parâmetros que eram capazes de realizar.

**Grupo exercícios gerais (GEG)**

O GEG realizará treinamento ao longo de 8 semanas, com dois atendimentos semanais, presencial e individualizado. Haverá um total de 16 atendimentos, cada um com duração de 50 a 60 minutos. O protocolo consistirá em aquecimento de 5 minutos de caminhada, alongamento e fortalecimento dos grupamentos musculares do tronco, quadril e membros inferiores. A progressão dos exercícios ocorrerá por meio de faixas elásticas, dos mais fáceis aos mais difíceis, e com adição de exercícios mais difíceis^23^. Na primeira semana não haverá uso de carga, porém a partir da segunda semana o GEG será testado com três cores diferentes de faixa de resistência elástica, da mais fácil à mais difícil e orientadas a realizar 3-5 repetições com cada faixa^23^. Elas decidirão qual faixa elástica consideram que seriam capazes de realizar 3 séries com 8-12 repetições, mantendo a qualidade do movimento^23^. As participantes escolherão a cor do elástico para cada exercício, e o elástico será posicionado acima da articulação do joelho. O teste de progressão da carga será realizado semanalmente e a progressão da carga será aumentada de acordo com a escala de Borg modificada (0-10), quando 3 (moderado) ou escores menores (fácil) forem alcançados, a progressão da carga será feita para os subsequentes elásticos^23^. O descanso entre os exercícios será de um minuto^28^. Quanto à evolução dos exercícios, caso as participantes não consigam progredir por dores suficientes para desistir do exercício, ou dificuldade em realizar pelo menos 8 repetições com qualidade, elas permanecerão com os parâmetros que eram capazes de realizar.

**Análise estatística**

A análise estatística será realizada no SPSS (Inc., Chicago, EUA). Os dados serão avaliados por meio de técnicas estatísticas exploratórias. Primeiramente, serão verificadas a normalidade e homogeneidade dos dados e, em seguida, serão adotadas as análises estatísticas adequadas para as variáveis. As diferenças entre os grupos e seus respectivos intervalos de confiança em 95% serão calculados pela construção de modelos lineares mistos de interação de dados de grupo em função do tempo. O modelo linear de efeito misto será aplicado aos resultados primários e secundários. “Tempo” e “grupo” serão considerados efeitos fixos, enquanto os participantes serão considerados efeitos aleatórios. Os valores da linha de base da variável dependente serão incluídos como uma covariável para a correção de possíveis diferenças de linha de base entre os grupos. A análise estatística seguirá o conceito de intenção de tratar.

Uma análise de mediação causal será conduzida usando o pacote “mediate” em R (The R Foundation for Statistical Computing). Uma abordagem de inferência baseada em modelo será usada para estimar o efeito médio da mediação causal, o efeito direto médio e o efeito total médio^29^.

**DISCUSSÃO**

Este estudo tem como objetivo avaliar se haverá diferença na intensidade da dor do quadril entre indivíduos com SDTM que se submeteram a um protocolo de exercícios com ênfase no treinamento do controle motor e aqueles que se submeteram a um programa de exercícios gerais não específicos. Considerando que nenhum estudo utilizou o treinamento neuromuscular como estratégia de tratamento da SDTM e não há evidências suficientes sobre a influência dessa intervenção nos aspectos clínico e biomecânico, torna-se necessário este estudo. O estudo utilizará dois protocolos diferentes de fácil aplicação clínica, com exercícios e orientações bem descritos, e equipamentos simples e de fácil acesso, para que as participantes possam adotar esses programas de autogestão.

Os exercícios são considerados fundamentais do tratamento não cirúrgico da dor musculoesquelética crônica^17^. Escolhemos dois tipos de protocolos: o GCM foi desenvolvido para ser específico para o grupo muscular alvo, com foco não apenas no fortalecimento, mas também na melhoria dos padrões de marcha, mantendo padrões de movimento corretos e eficientes e fornecendo orientações sobre como evitar posições agravantes como adução excessiva do fêmur durante as atividades funcionais. Para o GEG, será aplicado um protocolo de exercícios geral e não específico, sem orientação sobre posicionamento ou estratégias para evitar o agravamento da dor - serão realizados apenas exercícios de aquecimento, alongamento e fortalecimento não específico. Essas diferentes abordagens serão comparadas para determinar quaisquer diferenças em nossas medidas de resultados primários de dor do quadril, e se há algum efeito nas medidas de resultados físicos e psicológicos secundários.

SDTM é, por definição, uma síndrome dolorosa, portanto, a causa específica da dor pode ser difícil de definir, visto que mais de uma patologia pode apresentar sintomas semelhantes na região do quadril^5,6,17,19^. Aplicaremos uma combinação de múltiplos testes clínicos para evitar a inclusão de participantes que não possuem SDTM, por exemplo, osteoartrite de quadril^17,19^. O diagnóstico da SDTM é clínico e dois estudos de Grimaldi et al. 2017 e Ganderton et al. 2017 demonstraram que a palpação sobre o trocanter maior tem uma sensibilidade de 80% e 85,7%, respectivamente, e uma especificidade de 47% e 61,1%, respectivamente. Também relataram razões de verossimilhança de 0,43 e 2,2, respectivamente, demonstrando que esse teste sozinho, quando negativo, é capaz de descartar a presença de SDTM^6,19^. No entanto, a combinação da palpação e um dos outros testes FABER, apoio unipodal, FADER, FADER-R, ADD, ADD-R aumenta as chances de detecção da SDTM devido à especificidade desses testes, que pode chegar a 100%^19^.

A fim de manter a qualidade metodológica, este ensaio clínico estará em conformidade com os padrões do grupo consort^30^. As participantes do estudo serão randomizadas para participar de um dos grupos por meio de alocação oculta^17,30^. Devido à natureza da intervenção, não é possível cegar o clínico ou a participante para a alocação do grupo. Apenas o avaliador das medidas de resultado pode ser cego para a alocação do tratamento. A importância de não revelar nada a este avaliador sobre a natureza do seu tratamento será fortemente enfatizada às participantes. A análise estatística será conduzida às cegas para a alocação do grupo de tratamento - os grupos reais só serão revelados após a análise. Além disso, será utilizada a análise de intenção de tratar, que preserva a randomização do estudo e simula a situação da vida real, onde nem todos as participantes são submetidos ao tratamento completo^31^. Como limitação do estudo, destaca-se a participação apenas de mulheres, portanto, deve-se ter cuidado ao extrapolar os dados para homens com as mesmas condições.

A ideia da pesquisa surgiu a partir de estudos que apontavam os exercícios físicos como primeira linha de tratamento para o manejo das tendinopatias em membros superiores e inferiores, como a SDTM^5,12,17^. O exercício geral tem a capacidade de diminuir a entrada aferente nociceptiva para o sistema nervoso central e, assim, diminuir a dor^10^, no entanto, nosso protocolo de controle motor consiste, além de exercícios isotônicos, de exercícios isométricos que foram relatados como importantes para a melhora da dor periférica e central, liberando inibição cortical e reduzindo a dor no tendão^32^. Além disso, o grupo controle motor consiste no treinamento neuromuscular, com correção e manutenção do alinhamento dinâmico dos membros inferiores durante o treinamento da marcha, o que pode ser o motivo deste protocolo ser superior ao GEG. As descobertas deste estudo contribuirão para determinar o efeito sobre a dor do quadril de ambos GCM e GEG no tratamento de indivíduos com SDTM. Essas informações podem ser utilizadas pelos profissionais de saúde para auxiliá-los na tomada de decisão clínica e na seleção do programa de treinamento mais adequado para o manejo da SDTM.

**CRONOGRAMA DE EXECUÇÃO**

**
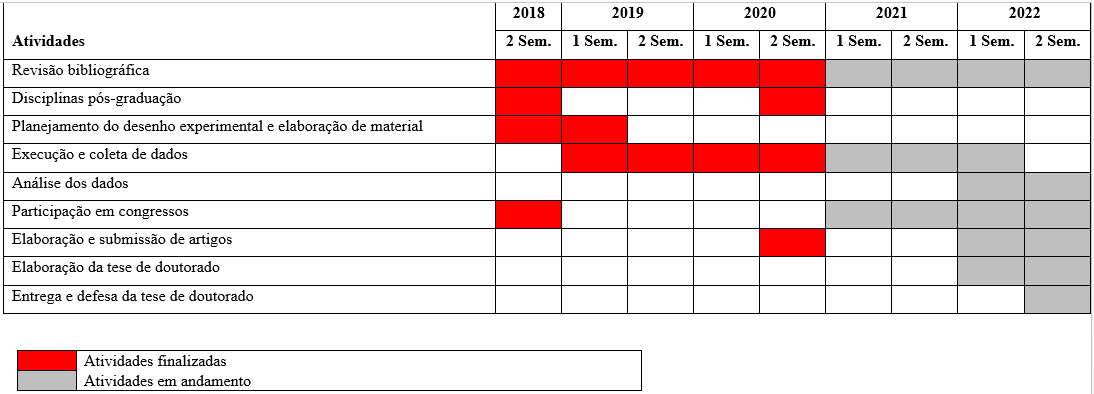
**

**REFERÊNCIAS**

1. Ho GWK, Howard TM. Greater trochanteric pain syndrome: More than bursitis and iliotibial tract friction. *Curr Sports Med Rep*. 2012;11(5):232-238. doi:10.1249/JSR.0b013e3182698f47

2. Reid D. The management of greater trochanteric pain syndrome: A systematic literature review. *J Orthop*. 2016;13(1):15-28. doi:10.1016/j.jor.2015.12.006

3. Williams BS, Cohen SP. Greater trochanteric pain syndrome: A review of anatomy, diagnosis and treatment. *Anesth Analg*. 2009;108(5):1662-1670. doi:10.1213/ane.0b013e31819d6562

4. Grimaldi A, Mellor R, Hodges P, Bennell K, Wajswelner H, Vicenzino B. Gluteal Tendinopathy: A Review of Mechanisms, Assessment and Management. *Sport Med*. 2015;45(8):1107-1119. doi:10.1007/s40279-015-0336-5

5. Ganderton C, Semciw A, Cook J, Pizzari T. Does menopausal hormone therapy (MHT), exercise or a combination of both, improve pain and function in post-menopausal women with greater trochanteric pain syndrome (GTPS)? A randomised controlled trial. *BMC Womens Health*. 2016;16(1):32. doi:10.1186/s12905-016-0311-9

6. Ganderton C, Semciw A, Cook J, Pizzari T. Demystifying the Clinical Diagnosis of Greater Trochanteric Pain Syndrome in Women. *J Women’s Heal*. 2017;26(6):633-643. doi:10.1089/jwh.2016.5889

7. Fearon AM, Cook JL, Scarvell JM, Neeman T, Cormick W, Smith PN. Greater Trochanteric Pain Syndrome Negatively Affects Work, Physical Activity and Quality of Life: A Case Control Study. *J Arthroplasty*. 2014;29(2):383-386. doi:10.1016/j.arth.2012.10.016

8. Ganderton C, Pizzari T, Harle T, Cook J, Semciw A. A comparison of gluteus medius , gluteus minimus and tensor facia latae muscle activation during gait in post-menopausal women with and without greater trochanteric pain syndrome q. *J Electromyogr Kinesiol*. 2017;33:39-47. doi:10.1016/j.jelekin.2017.01.004

9. Mellor R, Bennell K, Grimaldi A, et al. Education plus exercise versus corticosteroid injection use versus a wait and see approach on global outcome and pain from gluteal tendinopathy: Prospective, single blinded, randomised clinical trial. *Br J Sports Med*. 2018;52(22):1464-1472. doi:10.1136/bjsports-2018-k1662rep

10. Ganderton C, Semciw A, Cook J, Moreira E, Pizzari T. Gluteal Loading Versus Sham Exercises to Improve Pain and Dysfunction in Postmenopausal Women with Greater Trochanteric Pain Syndrome: A Randomized Controlled Trial. *J Women’s Heal*. 2018;27(6):815-829. doi:10.1089/jwh.2017.6729

11. Rompe JD, Segal NA, Cacchio A, Furia JP, Morral A, Maffulli N. Home Training, Local Corticosteroid Injection, or Radial Shock Wave Therapy for Greater Trochanter Pain Syndrome. *Am J Sports Med*. 2009;37(10):1981-1990. doi:10.1177/0363546509334374

12. Barratt PA, Brookes N, Newson A. 1Conservative treatments for greater trochanteric pain syndrome: a systematic review. *Br J Sports Med*. 2017;51(2):97-104. doi:10.1136/bjsports-2015-095858

13. Allison K, Salomoni SE, Bennell KL, et al. Hip abductor muscle activity during walking in individuals with gluteal tendinopathy. *Scand J Med Sci Sports*. 2018;28(2):686-695. doi:10.1111/sms.12942

14. Allison K, Vicenzino B, Wrigley T V., Grimaldi A, Hodges PW, Bennell KL. Hip Abductor Muscle Weakness in Individuals with Gluteal Tendinopathy. *Med Sci Sports Exerc*. 2016;48(3):346-352. doi:10.1249/MSS.0000000000000781

15. Allison K, Wrigley T V., Vicenzino B, Bennell KL, Grimaldi A, Hodges PW. Kinematics and kinetics during walking in individuals with gluteal tendinopathy. *Clin Biomech*. 2016;32:56-63. doi:10.1016/j.clinbiomech.2016.01.003

16. Tegner Y, Lysholm J. Rating systems in the evaluation of knee ligament injuries. *Clin Orthop Relat Res*. 1985;(198):43-49. http://www.ncbi.nlm.nih.gov/pubmed/4028566.

17. Mellor R, Grimaldi A, Wajswelner H, et al. Exercise and load modification versus corticosteroid injection versus ‘wait and see’ for persistent gluteus medius/minimus tendinopathy (the LEAP trial): a protocol for a randomised clinical trial. *BMC Musculoskelet Disord*. 2016;17(1):196. doi:10.1186/s12891-016-1043-6

18. Fearon AM, Scarvell JM, Neeman T, Cook JL, Cormick W, Smith PN. Greater trochanteric pain syndrome: defining the clinical syndrome. *Br J Sports Med*. 2013;47(10):649-653. doi:10.1136/bjsports-2012-091565

19. Grimaldi A, Mellor R, Nicolson P, Hodges P, Bennell K, Vicenzino B. Utility of clinical tests to diagnose MRI-confirmed gluteal tendinopathy in patients presenting with lateral hip pain. *Br J Sports Med*. 2017;51(6):519-524. doi:10.1136/bjsports-2016-096175

20. Speers CJ, Bhogal GS. Greater trochanteric pain syndrome: a review of diagnosis and management in general practice. *Br J Gen Pract*. 2017;67(663):479-480. doi:10.3399/bjgp17X693041

21. Ostelo RWJG, de Vet HCW, Vlaeyen JWS, et al. Behavioral Graded Activity Following First-Time Lumbar Disc Surgery. *Spine (Phila Pa 1976)*. 2003;28(16):1757-1765. doi:10.1097/01.BRS.0000083317.62258.E6

22. Garcia SC, Dueweke JJ, Mendias CL. Optimal Joint Positions for Manual Isometric Muscle Testing. *J Sport Rehabil*. 2016;25(4). doi:10.1123/jsr.2015-0118

23. Picha KJ, Almaddah MR, Barker J, Ciochetty T, Black WS, Uhl TL. Elastic Resistance Effectiveness on Increasing Strength of Shoulders and Hips. *J Strength Cond Res*. 2019;33(4):931-943. doi:10.1519/JSC.0000000000002216

24. Zapparoli FY, Riberto M. Isokinetic Evaluation of the Hip Flexor and Extensor Muscles: A Systematic Review. *J Sport Rehabil*. 2017;26(6):556-566. doi:10.1123/jsr.2016-0036

25. Sehn F, Chachamovich E, Vidor LP, et al. Cross-Cultural Adaptation and Validation of the Brazilian Portuguese Version of the Pain Catastrophizing Scale. *Pain Med*. 2012;13(11):1425-1435. doi:10.1111/j.1526-4637.2012.01492.x

26. Siqueira FB, Teixeira-Salmela LF, Magalhães L de C. Análise das propriedades psicométricas da versão brasileira da escala tampa de cinesiofobia. *Acta Ortopédica Bras*. 2007;15(1):19-24. doi:10.1590/S1413-78522007000100004

27. Caumo W, Antunes L, Lorenzzi Elkfury J, et al. The Central Sensitization Inventory validated and adapted for a Brazilian population: psychometric properties and its relationship with brain-derived neurotrophic factor. *J Pain Res*. 2017;Volume 10:2109-2122. doi:10.2147/JPR.S131479

28. Kraemer WJ, Adams K, Cafarelli E, et al. Progression models in resistance training for healthy adults. *Med Sci Sports Exerc*. 2002;34(2):364-380. doi:10.1097/00005768-200202000-00027

29. Tingley D, Yamamoto T, Hirose K, Keele L, Imai K. mediation : R Package for Causal Mediation Analysis. *J Stat Softw*. 2014;59(5). doi:10.18637/jss.v059.i05

30. Altman DG. The Revised CONSORT Statement for Reporting Randomized Trials: Explanation and Elaboration. *Ann Intern Med*. 2001;134(8):663. doi:10.7326/0003-4819-134-8-200104170-00012

31. Elkins MR, Moseley AM. Intention-to-treat analysis. *J Physiother*. 2015;61(3):165-167. doi:10.1016/j.jphys.2015.05.013

32. Rio E, Kidgell D, Purdam C, et al. Isometric exercise induces analgesia and reduces inhibition in patellar tendinopathy. *Br J Sports Med*. 2015;49(19):1277-1283. doi:10.1136/bjsports-2014-094386

33. Paiva E, Azevedo D, Pereira A, et al. Measurement properties of instruments used to assess patients with Greater Trochanteric Pain Syndrome (Under Review)

34. Costa RM de P, Cardinot TM, Mathias LNCDC, Leporace G, de Oliveira LP. Validation of the Brazilian version of the Hip Outcome Score (HOS) questionnaire. Adv Rheumatol [Internet]. 2018 Dec 24;58(1):4. Available from: https://advancesinrheumatology.biomedcentral.com/articles/10.1186/s42358-018-0007-y

35. Polesello GC, Godoy GF, Trindade CA de C, de Queiroz MC, Honda E, Ono NK. Translation and cross-cultural adaptation of the International Hip Outcome Tool (iHOT) into Portuguese. Acta Ortop Bras. 2012;20(2):88-92B.
