# Supplementary material for: Motor control exercises versus general exercises for greater trochanteric pain syndrome: A protocol of a randomized controlled trial
Source: PLoS One. 2022 Jun 24;17(6):e0269230. doi: 10.1371/journal.pone.0269230 (PMC9231741; doi:10.1371/journal.pone.0269230)
Supplement: S2 Protocol — (DOCX) [file pone.0269230.s003.docx]

**INTRODUCTION**

Greater trochanter pain syndrome (GTPS) is a broad term used to define pain and tenderness in the greater trochanter region of the femur^1,2^. It is estimated that 10 to 25% of the population will develop some type of pain in the lateral region of the hip^1–3^, predominantly in women over 40 years old ^2–6^. The GTPS directly impacts the worsening of quality of life, which is similar to the findings in individuals with severe hip osteoarthritis^7^. Women have mechanisms that can influence the onset of GTPS, such as smaller gluteal tendon insertions in the femur, shorter gluteal moment arm and gluteus medius weakness^6^.

Changes in the function of the gluteus medius and minimus muscles can lead to poor hip and pelvic control and lead to increased hip adduction in the frontal plane, especially in unilateral support positions, such as in gait^6,8^. The literature indicates that active treatments, those in which participants perform physical exercises, are preferable to passive approaches in the treatment of GTPS. Active treatments result in better results in the short^9,10^, medium^9^and long term^9-11^in terms of pain and function when compared to rest, shock wave therapy and/or invasive treatments, eg, injection. corticosteroid^5,9,11^. Surgical interventions are generally reserved for recalcitrant cases^1,2,5,12^.

Despite previous knowledge that physical exercise generates benefits for GTPS and chronic pain in general, no study has attempted to demonstrate the efficacy of motor control exercise for GTPS. Individuals with GTPS have abnormal control of lower limb movements and deficient neuromuscular parameters^8,13,14^and alterations in the trunk and pelvic kinematics during walking^15^, however, no study used neuromuscular training as a treatment strategy and there is insufficient evidence about the influence of this intervention on the clinical and biomechanical aspects of these participants. Thus, this study aims to compare the effect of a general exercise protocol versus a motor control training program on the onset and after treatment at 8 and 60 weeks in women with GTPS.

Our hypothesis is that both women undergoing the motor control protocol and women undergoing the general exercise protocol will show improvements in all outcomes evaluated, although the motor control group is superior because the intervention is specific to the hip region.

**MATERIALS AND METHODS**

This is a randomized controlled trial, blind to evaluator and statistician, with 2 arms, registered in the Brazilian Registry of Clinical Trials (RBR-37gw2x) and has a Universal Trial Number (U1111-1234-0705)

**Ethical approval and consent**

The study was approved by the Research Ethics Committee of the Faculty of Philosophy and Sciences, São Paulo State University (UNESP) , Campus of Marília (CAAE: 87372318.1.0000.5406). All participants will sign an informed consent form.

**Participants**

**Recruitment**

Women with GTPS will be recruited from the community, universities and within the public health service, through digital means (facebook and instagram) and written dissemination (flyers). Interested participants will contact the principal investigator by phone or social media.

**Clinical screening**

**Screening by phone**

Initial screening will be done by phone. Potential participants will be eligible for the study if they are between 18 and 70 years old, do not engage in regular physical activity (Tegner activity level ≤4) ^16^and have had lateral hip pain for ≥ 3 months. They should have pain during one or more of the seven daily activities: walking, standing for a long period, getting up from a sitting position, sitting for a long time, going up and down stairs, going up and down hills and lying down. side. Potential participants will be ineligible for the study if they have a body mass index (BMI) > 36 kg/m2, have received some type of invasive intervention for lateral hip pain or physical therapy intervention for hip pain in the past 12 weeks, have morning hip stiffness ≤ 60 minutes, any disease that affects the neuromuscular system or that may prevent data collection, has had spine or hip surgery, any infectious condition, any neoplasm, or cannot commit to participate during treatment^10-12,15,17^.

**Physiotherapeutic evaluation**

Potential participants who meet the eligibility criteria over the phone will undergo two assessments. The first will be held to screen for inclusion and exclusion criteria that were not possible to assess by phone, as well as explain in more detail the nature of the study and the commitment required, with an opportunity for participants to ask questions, and the second will be carried out by a blind evaluator to assess outcomes. The physical therapy evaluation will be carried out at São Paulo State University and will take about 40 minutes to 1 hour. The assessment will be done by a physical therapist with 8 years of experience, with a master's degree in the area of ​​musculoskeletal disorders. Demographic data will be collected, including age, weight, height, BMI, etc. It will be determined whether the participants have the ability to manipulate shoes and socks^18^, have limited range of motion of the spine, hips or lower limbs that affect gait or data collection, or a visible discrepancy in the lower limbs. It will be evaluated whether the participants have sensitivity to palpation over the greater trochanter region of the femur^8,19,20^. If both hips are symptomatic, the more painful side will be evaluated ^10^. The Trendelenburg sign, which is the pelvic drop during unipodal support, indicating inefficiency of the hip abductor muscles, will be evaluated^5,8,19^.

The tests that will be used for clinical evaluation aim to define the exact location of the pain and transmit compressive and/or tension forces in the tendons of the hip abductor muscles on the greater trochanter. Participants must experience pain over the greater trochanter of the femur in ≥ two of the seven provocative clinical trials for pain^12,14,17^. They are:

• Greater trochanter palpation: positive palpation sensitivity, considered when there is pain over the greater trochanter of the femur, during assessment in lateral decubitus (LD) with the pain side up, flexion of the hip joint and knees together^5,6,8.11.17^. This test has been reported to have a sensitivity of 85.7% and a specificity of 61.1%^6^.

• FADER: Participants in dorsal decubitus (DD) should report pain over the greater trochanter when the affected lower limb is positioned in 90º flexion, adduction and external rotation of the hip^17^.

• FADER-R with static muscle test: Participants in DD, have to report pain when the affected lower limb is positioned in the same way, but maintaining isometric resistance to internal rotation at the end of the range of motion^5,6,8,11,17^. This test is a modification of the resisted external rotation test, which has been reported to have 42.3% sensitivity and 95% specificity^6^.

• FABER: Participants in DD should report pain when the affected lower limb is positioned in flexion, abduction and external rotation of the hip^6,17,18^. This test has been reported to have a sensitivity of 50% and a specificity of 83%^18^.

• ADD: Participants must report pain when the affected lower limb is positioned in passive hip adduction in LD^17^.

• ADD R: Participants must report pain when the affected lower limb is positioned in passive hip adduction in LD, but maintaining isometric resistance in abduction^17^. This test has been reported to have a sensitivity of 50% and a specificity of 97.3%^6^.

• Single leg Positioning: The participants should report pain to lean on one leg for 30 seconds. This test has a sensitivity of 45.4% and a specificity of 84.2% to indicate SDTM^6^.

**Sample size and power analysis**

Sample calculation was performed using the G * Power software, based on the measure of the level of pain, as it is the primary outcome of the research. A power of 0.80, error probability α 0.05, effect size of 0.5 and dropout rate of 15% were used, for this we will recruit 60 participants (30 in each group)^9^.

**Randomization, allocation and blinding**

If all eligibility criteria are met, and the presence of GTPS is determined, participants will be randomly assigned to receive 1) motor control exercises or 2) general exercises. Participants will be randomly allocated to two therapeutic arms using permuted block randomization to balance the number of patients allocated to each group. The randomization sequence of the permuted block (with six patients per block) will be generated by the website www.sealedenvelope.com. Participants will be informed of their random allocation by one of the researchers not involved in the evaluation process. After randomization, participants will be invited to return for physical therapy assessment, with a blind assessor, a physical therapist with 8 years of experience and a doctorate in the area of ​​musculoskeletal disorders. This evaluator will not participate in the screening or interventions. All participants will be advised not to disclose to the evaluator any details about the intervention program they have received (to ensure the allocation is hidden). In this evaluation, participants will give their free and informed consent.

**Outcome assessment**

**Primary outcome**

There is one primary outcome measure: 1) Average pain in the previous week.

1) Pain intensity will be assessed at baseline and after treatment in 8 weeks. They will also be evaluated at 60 weeks, which is a secondary time point for the primary outcome. Pain intensity will be assessed using the visual analogue scale (VAS)^5^. Formed by a 100 mm horizontal line, anchored by the words “no pain/discomfort” and “worst imaginable pain/discomfort”^5^. To measure pain, the participant will be asked to indicate the level of pain they are currently feeling and the average pain they felt in the last week, marking the scale with a line. A ruler will be used to measure the obtained value^5^.

**Secondary outcomes**

There are seven secondary outcome measures: 1) Perceived Global Effect, 2) Muscle Strength, 3) Pain Catastrophizing, 4) Kinesiophobia, 5) Central Sensitization, 6) Muscle Recruitment, and 7) Hip Functionality.

1) The global perceived effect scale (GPE) will be used after treatment at 8 and 60 weeks^21^. This is a 7-point scale (1=completely recovered, 7=worse than ever) to assess recovery. GPE assesses the participant's perception regarding the modification of their clinical condition after the intervention, and is evaluated by a simple and easy-to-understand question with alternative answer options that will be dichotomized into "improved" ("completely recovered" and "much better ") versus “not improved” (“slightly improved”, “not changed”, “slightly worse”, “very much worse”, “worse than ever”^21^.

2) Isometric strength of the hip abductor and extensor muscles will be assessed at baseline and after treatment, 8 weeks during post-intervention reassessment. A manual dynamometer model Lafayette (Lafayette Instruments) will be used, which has proven to be a valid method for evaluating isolated muscle contraction strength^22^. To assess the hip abductor musculature, participants will be placed in the supine position on a stretcher, stabilized by velcro bands around the pelvis and above the lateral ankle malleolus to avoid compensatory movements and the influence of the assessor resistance^22^. The dynamometer will be positioned above the lateral ankle malleolus and fixed to the stretcher by an inelastic strap, it will be positioned without rotation, with 10º of hip abduction to minimize the potential for compression of the tendons against the greater trochanter^14^. The untested lower limb will be positioned with 45º of hip and knee flexion, with the foot positioned on the stretcher and the upper limbs supported by the side of the body (figure 2)^14^. For evaluation of the hip extensors, participants will be positioned in the prone position with knee flexion on the lower limb to be evaluated. The dynamometer will be positioned on the posterior region of the thigh, above the popliteal fossa and will be stabilized by an inelastic velcro strap^23^. Another Velcro strap will be placed around the pelvis (figure 3)^23^. The measurement of limb length will be performed with a measuring tape to calculate torque, from the greater trochanter to the center of the dynamometer, and will be recorded in meters^23,24^. The maximum voluntary contraction tests will be performed 3 times with 5 seconds of duration for each contraction, with 30 seconds of rest between each attempt^23^. Participants will be verbally encouraged to perform as much strength as possible during the test. The strength values ​​will be normalized by the weight of each participant^23^.

Figure 2. Assessment of isometric strength of hip abductor muscles


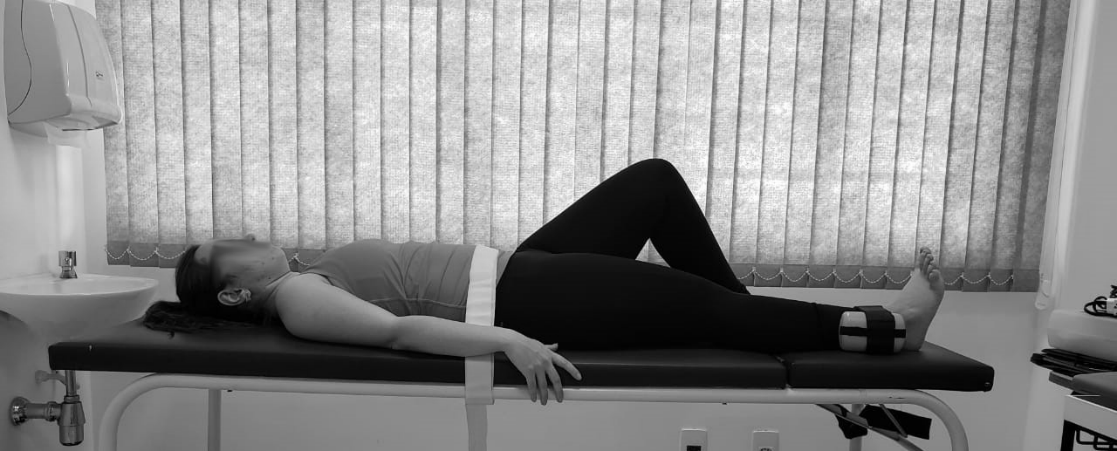


Figure 3. Evaluation of isometric strength of hip extensors muscles


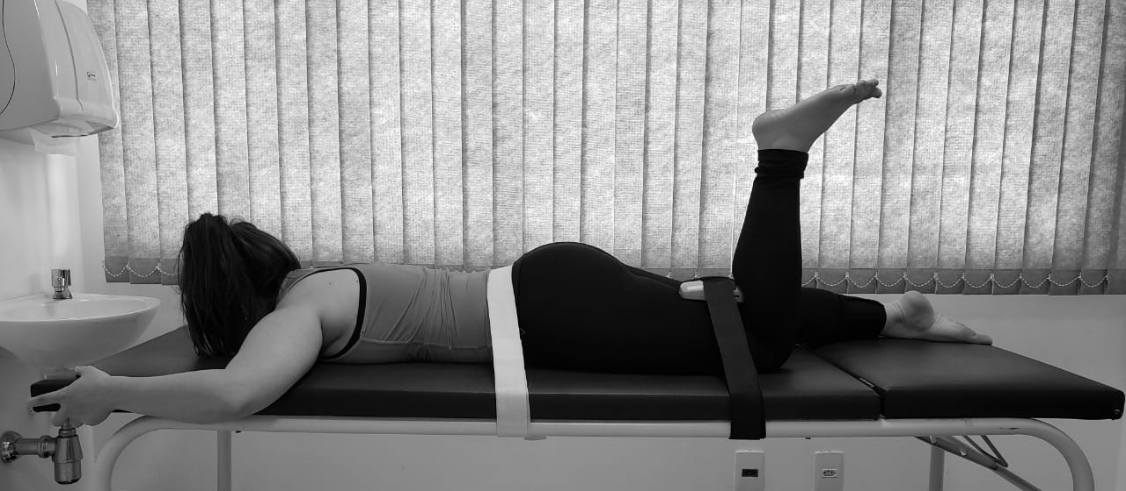


3) Pain catastrophizing will be measured with the Pain Catastrophizing Scale (PCS) at baseline and after treatment, at 8 and 60 weeks during post-intervention reassessment. The PCS is a validated^25^, self-administered, 13-item questionnaire that assesses catastrophic thoughts, feelings and behavior when in pain^25^. This questionnaire assesses three main domains: helplessness, magnification and rumination in relation to pain. Results are calculated as the sum of all survey items and total scores range from 0 to 52, with higher scores indicating higher levels of pain catastrophizing^25^.

4) Kinesiophobia will be assessed with the Tampa scale of kinesiophobia, which is a validated, self-administered questionnaire with 17 questions and translated into Portuguese. Kinesiophobia will be assessed at baseline and after treatment, at 8 and 60 weeks during post-intervention reassessment. The final score can be at least 17 and at most 68 points, with higher scores indicating higher degrees of kinesiophobia^26^.

5) Central sensitization will be measured with the central sensitization inventory, which is a self-administered health symptoms questionnaire designed as an easy-to-administer tracker for patients at high risk of central sensitization or to assess symptoms related to central sensitization^27^. Central sensitization will be measured at baseline and after treatment at 8 and 60 weeks during post-intervention reassessment. It has also been recommended as a component of an algorithm to help classify chronic pain patients with central sensitization and to help differentiate them from patients with primary neuropathic and nociceptive pain^27^.

6) Muscle recruitment during treadmill walking (MOVEMENT^®^) will be assessed at baseline and after treatment at 8 weeks follow-up. Surface electromyography will be used in three different walking conditions: preferential speed, top speed but no running and 3.0km/h. Each speed will be recorded for one minute. The familiarization with the treadmill will be carried out for 5 minutes at the self-selected speed, the interval between each test of each speed will be 1 minute and the participants will take the test barefoot^13^. The electromyographic signals will be captured using an 8-channel biological signal acquisition model module (New Miotool Wireless, Porto Alegre, Brazil) with 2000 gain, 16-bit A/D converter, 1010 Ohm / 2 pF input impedance , common mode rejection ratio 126 dB, bandpass filter 20–500 Hz and sampling rate 2 kHz. In addition, the software allows real-time filming via the webcam. All wires will be joined and secured to avoid motion artifact. The muscles of each participant will be located, according to the SENIAM group, and the electrodes will be placed unilaterally on the affected side (or more painful if bilateral) and longitudinally oriented with the muscle fibers of the muscle to be evaluated^10,13^. Trichotomy will be performed in the area of ​​electrode placement and skin abrasion with gauze to reduce impedance, in addition to the use of alcohol to clean the skin^13,14,15^. Active bipolar electrodes will be used, which will be placed in a bipolar configuration, with a capture area of ​​1cm in diameter and a distance of 2cm between them^13,14^. A reference electrode will be placed over the ulnar styloid process. The muscles evaluated are:

- Gluteus medius (GMED): electrodes will be placed 50% along a line from the iliac crest to the most lateral point of the greater trochanter.
- Gluteus maximus (GMAX): The electrodes will be placed 50% along a line between the sacral vertebrae and the most lateral point of the greater trochanter. This position corresponds to the greatest prominence of the middle of the buttocks, well above the visible bulge of the greater trochanter.
- Tensor fascia latae (TFL): The electrodes will be placed proximally 1/6 of the way along a line from the anterosuperior iliac spine (ASIS) to the lateral femoral condyle.

7) Hip functionality will be measured using the Victorian Institute of Sport Assessment questionnaire for gluteal tendinopathy (VISA-G) and will be assessed at baseline and after treatment, 8 and 60 weeks during post-intervention reassessment. This is a self-administered questionnaire, translated, culturally adapted and validated for the Portuguese Brasileiro^33^. Visa-G is specific to assess the severity of deficiency in people with gluteal tendinopathy. It is an 8-item questionnaire that assesses pain and function. Scores range from 0 to 100 and higher values ​​indicate less pain and better functionality^33^.

7) Hip-related disability will be measured with the Hip Outcome Score (HOS), which is a self-administered questionnaire to assess young, physically active or both patients with hip disorders. It consists of 28 items divided into two subscales: activities of daily living and sports activities. The total score for each subscale ranges from 0 to 100, where higher scores denote better hip function. The scores for each subscale were calculated separately^34^.

7) Disability will also be assessed using the International Hip Outcome Tool (iHOT) which is a 12-question self-administered questionnaire. The questions are evaluated using a visual analogue scale, so each question has a 10 cm line and the participants must add a vertical line crossing the horizontal line, and the farther to the left, the worse the symptoms. The result of each question can vary between 0 and 100 and to determine the result, all the questions must be added and divided by the number of answered questions^35^.

**Analysis of electromyographic data**

The electromyographic analysis will be performed using Matlab® software. The fourth-order Butterworth high-pass filter with a cut-off frequency of 20 Hz, the fourth-order Butterworth low-pass filter with a cut-off frequency of 500 Hz, full-wave signal rectification and a low-pass filter of 6 Hz will form the linear envelope. The values ​​of the linear envelope of the muscles will be normalized by the highest value obtained in the maximum voluntary isometric contraction (MVIC) in the assessment and reassessment of their respective muscles.

**Interventions**

**Motor Control Group (MCG)**

The motor control program will be carried out over 8 weeks, with two weekly appointments, face-to-face and individualized. There will be a total of 16 sessions, each lasting from 50 to 60 minutes. The protocol will consist of isotonic and isometric strengthening exercises, focusing on the abductor and hip extensor muscles with coordination through verbal commands to improve dynamic motor control of the lower limbs. Exercise progression will occur through elastic bands, from the easiest to the most difficult, and with the addition of more difficult exercises^23^. In the first week there will be no use of loads, but from the second week on, the MCG will be tested with three different colors of elastic bands, from the easiest to the most difficult, and they will be instructed to perform 3-5 repetitions with each band^23^. They will decide which elastic band they feel would be able to perform 3 sets with 8-12 repetitions, maintaining movement quality^23^. The participants will choose the elastic color for each exercise and the elastic will be positioned above the knee joint. The load progression test will be performed weekly and the load progression will be increased according to the modified Borg scale (0-10), when 3 (moderate) or lower scores (easy) are reached, the load progression will be performed for the elastic subsequent^23^. Rest between exercises will be one minute^28^.

As for the evolution of the exercises, if the participants are unable to progress due to enough pain to give up the exercise, or difficulty in performing at least 8 repetitions with quality, they will remain with the parameters they were able to perform.

**General exercises group (GEG)**

GEG will carry out training over 8 weeks, with two weekly sessions, face-to-face and individualized. There will be a total of 16 sessions, each lasting from 50 to 60 minutes. The protocol will consist of a 5-minutes walking warm-up, stretching and strengthening the muscle groups of the trunk, hip and lower limbs. Exercise progression will occur through elastic bands, from the easiest to the most difficult, and with the addition of more difficult exercises^23^. In the first week there will be no use of loads, but from the second week on, the GEG will be tested with three different colors of elastic resistance range, from the easiest to the most difficult and oriented to perform 3-5 repetitions with each range^23^. They will decide which elastic band they consider would be capable of performing 3 sets with 8-12 repetitions, maintaining movement quality^23^. Participants will choose the rubber band color for each exercise, and the band will be positioned above the knee joint. The load progression test will be performed weekly and the load progression will be increased according to the modified Borg scale (0-10), when 3 (moderate) or lower scores (easy) are reached, the load progression will be performed for subsequent elastics^23^. Rest between exercises will be one minute^28^. As for the evolution of the exercises, if the participants are unable to progress due to enough pain to give up the exercise, or difficulty in performing at least 8 repetitions with quality, they will remain with the parameters they were able to perform.

**Statistical analysis**

The study will be run as a superiority trial. The statistical analysis will follow the intention-to-treat concept. Statistical analysis will be performed using the software IBM SPSS Statistics for Windows, version 20.0 (IBM Corp., Armonk, N.Y., USA) and statistician will be blind. Data will be evaluated using exploratory statistical techniques. Firstly, the normality and homogeneity of the data will be verified and then the appropriate statistical analyses will be adopted for the variables. The between-group differences and their respective confidence intervals at 95% will be calculated by constructing mixed linear models interaction of group data versus time. The linear mixed-effect model will be applied to the primary and secondary outcomes. “Time” and “group” will be considered fixed effects, whereas the participants will be considered random effect. The dependent variable baseline values will be included as a covariate for the correction of possible baseline between-group differences.

A causal mediation analysis will be conducted using the “mediate” package in R (The R Foundation for Statistical Computing). A model-based inference approach will be used to estimate the average causal mediation effect (ACME), average direct effect (ADE) and the average total effect^29^.

**DISCUSSION**

This study aims to assess whether there will be a difference in hip pain intensity between individuals with GTPS who underwent an exercise protocol with emphasis on motor control training and those who underwent a nonspecific general exercise program. Considering that no study used neuromuscular training as a treatment strategy for GTPS and there is insufficient evidence on the influence of this intervention on clinical and biomechanical aspects, this study is necessary. The study will use two different protocols that are easy to clinically apply, with simple and well-described exercises, accessible equipment, so that participants can adopt these self-management programs.

Exercises are considered the cornerstone nonsurgical treatment for chronic musculoskeletal pain^17^. We chose two types of protocols: the MCG was developed to be specific to the target muscle group, focusing not only on strengthening but also on improving gait patterns, maintaining correct and efficient movement patterns, and providing guidance on how to avoid aggravating positions as excessive adduction of the femur during functional activities. For the GEG, a general and non-specific exercise protocol will be applied, without guidance on positioning or strategies to avoid worsening pain - only non-specific warm-up, stretching and strengthening exercises will be performed. These different approaches will be compared to determine any differences in our primary hip pain outcome measures, and whether there is any effect on the secondary physical and psychological outcome measures.

GTPS is, by definition, a painful syndrome, therefore, the specific cause of pain may be difficult to define, as more than one pathology can present similar symptoms in the hip region^5,6,17,19^. We will apply a combination of multiple clinical tests to avoid including participants who do not have GTPS, eg hip osteoarthritis^17,19^. The diagnosis of GTPS is clinical and two studies by Grimaldi et al. 2017 and Ganderton et al. 2017 demonstrated that palpation over the greater trochanter has a sensitivity of 80% and 85.7%, respectively, and a specificity of 47% and 61.1%, respectively. They also reported likelihood ratios of 0.43 and 2.2, respectively, demonstrating that this test alone, when negative, is able to rule out the presence of GTPS^6,19^. However, the combination of palpation and one of the other tests, FABER, Single leg stance for 30 seconds , FADER, FADER-R, ADD, ADD-R increases the chances of detecting GTPS due to the specificity of these tests, which can reach 100%^19^.

In order to maintain methodological quality, this clinical trial will comply with the standards of the consort group^30^. Study participants will be randomized to join one of the groups through the concealed allocation^17.30^. Due to the nature of the intervention, it is not possible to blind the clinician or participant to group allocation. Only the assessor of outcome measures and the statistician are able to be blinded to treatment allocation. The importance of not revealing anything to the assessor about the nature of the treatment will be strongly emphasized to participants. Statistical analysis will be conducted blind to treatment group allocation - actual groups will only be revealed after analysis. In addition, intention-to-treat analysis will be used, which preserves the study's randomization and simulates the real-life situation^31^. As a limitation of the study, we highlight the participation of women only, so care must be taken when extrapolating data to men with the same conditions.

The research idea came from studies that indicated physical exercise as the first line of treatment for the management of tendinopathies in the upper and lower limbs, such as the GTPS^5,12,17^. General exercise has the ability to decrease nociceptive afferent input to the central nervous system and thus decrease pain^10^, however, our motor control protocol consists, in addition to isotonic exercises, of isometric exercises that have been reported as important for the improvement of the peripheral and central pain, cortical inhibition releasing and reducing pain in the tendon^32^. In addition, the MCG consists of neuromuscular training, with correction and maintenance of the dynamic alignment of the lower limbs during gait training, which may be the reason why this protocol is superior to GEG. The findings of this study will contribute to determine the effect on hip pain of both MCG and GEG in the treatment of individuals with GTPS. This information can be used by health professionals to assist them in clinical decision making and in selecting the most appropriate training program for the management of GTPS.

**REFERENCES**

1. Ho GWK, Howard TM. Greater trochanteric pain syndrome: More than bursitis and iliotibial tract friction. *Curr Sports Med Rep*. 2012;11(5):232-238. doi:10.1249/JSR.0b013e3182698f47

2. Reid D. The management of greater trochanteric pain syndrome: A systematic literature review. *J Orthop*. 2016;13(1):15-28. doi:10.1016/j.jor.2015.12.06

3. Williams BS, Cohen SP. Greater trochanteric pain syndrome: A review of anatomy, diagnosis and treatment. *Anaesth Analg*. 2009;108(5):1662-1670. doi:10.1213/ane.0b013e31819d6562

4. Grimaldi A, Mellor R, Hodges P, Bennell K, Wajswelner H, Vicenzino B. Gluteal Tendinopathy: A Review of Mechanisms, Assessment and Management. *Sport Med*. 2015;45(8):1107-1119. doi:10.1007/s40279-015-0336-5

5. Ganderton C, Semciw A, Cook J, Pizzari T. Does menopausal hormone therapy (MHT), exercise or a combination of both, improve pain and function in post-menopausal women with greater trochanteric pain syndrome (GTPS)? A randomized controlled trial. *BMC Womens Health*. 2016;16(1):32. doi:10.1186/s12905-016-0311-9

6. Ganderton C, Semciw A, Cook J, Pizzari T. Demystifying the Clinical Diagnosis of Greater Trochanteric Pain Syndrome in Women. *J Women's Heal*. 2017;26(6):633-643. doi:10.1089/jwh.2016.5889

7. Fearon AM, Cook JL, Scarvell JM, Neeman T, Cormick W, Smith PN. Greater Trochanteric Pain Syndrome Negatively Affects Work, Physical Activity and Quality of Life: A Case Control Study. *J Arthroplasty*. 2014;29(2):383-386. doi:10.1016/j.arth.2012.10.016

8. Ganderton C, Pizzari T, Harle T, Cook J, Semciw A. A comparison of gluteus medius, gluteus minimus and tensor facia latae muscle activation during gait in post-menopausal women with and without greater trochanteric pain syndrome q. *J Electromyogr Kinesiol*. 2017;33:39-47. doi:10.1016/j.jelekin.2017.01.004

9. Mellor R, Bennell K, Grimaldi A, et al. Education plus exercise versus corticosteroid injection use versus a wait and see approach on global outcome and pain from gluteal tendinopathy: Prospective, single blinded, randomized clinical trial. *Br J Sports Med*. 2018;52(22):1464-1472. doi:10.1136/bjsports-2018-k1662rep

10. Ganderton C, Semciw A, Cook J, Moreira E, Pizzari T. Gluteal Loading Versus Sham Exercises to Improve Pain and Dysfunction in Postmenopausal Women with Greater Trochanteric Pain Syndrome: A Randomized Controlled Trial. *J Women's Heal*. 2018;27(6):815-829. doi:10.1089/jwh.2017.6729

11. Disrupt JD, Segal NA, Cacchio A, Furia JP, Morral A, Maffulli N. Home Training, Local Corticosteroid Injection, or Radial Shock Wave Therapy for Greater Trochanter Pain Syndrome. *Am J Sports Med*. 2009;37(10):1981-1990. doi:10.1177/0363546509334374

12. Barratt PA, Brookes N, Newson A. 1Conservative treatments for greater trochanteric pain syndrome: a systematic review. *Br J Sports Med*. 2017;51(2):97-104. doi:10.1136/bjsports-2015-095858

13. Allison K, Salomoni SE, Bennell KL, et al. Hip abductor muscle activity during walking in individuals with gluteal tendinopathy. *Scand J Med Sci Sports*. 2018;28(2):686-695. doi:10.1111/sms.12942

14. Allison K, Vicenzino B, Wrigley TV., Grimaldi A, Hodges PW, Bennell KL. Hip Abductor Muscle Weakness in Individuals with Gluteal Tendinopathy. *Med Sci Sports Exercise*. 2016;48(3):346-352. doi:10.1249/MSS.0000000000000781

15. Allison K, Wrigley TV., Vicenzino B, Bennell KL, Grimaldi A, Hodges PW. Kinematics and kinetics during walking in individuals with gluteal tendinopathy. *Clin Biomech*. 2016;32:56-63. doi:10.1016/j.clinbiomech.2016.01.03

16. Tegner Y, Lysholm J. Rating systems on the evaluation of knee ligament injuries. *Clin Orthop Relat Res*. 1985;(198):43-49. http://www.ncbi.nlm.nih.gov/pubmed/4028566.

17. Mellor R, Grimaldi A, Wajswelner H, et al. Exercise and load modification versus corticosteroid injection versus 'wait and see' for persistent gluteus medius/minimus tendinopathy (the LEAP trial): a protocol for a randomized clinical trial. *BMC Musculoskelet Disord*. 2016;17(1):196. doi:10.1186/s12891-016-1043-6

18. Fearon AM, Scarvell JM, Neeman T, Cook JL, Cormick W, Smith PN. Greater trochanteric pain syndrome: defining the clinical syndrome. *Br J Sports Med*. 2013;47(10):649-653. doi:10.1136/bjsports-2012-091565

19. Grimaldi A, Mellor R, Nicolson P, Hodges P, Bennell K, Vicenzino B. Utility of clinical tests to diagnose MRI-confirmed gluteal tendinopathy in patients presenting with lateral hip pain. *Br J Sports Med*. 2017;51(6):519-524. doi:10.1136/bjsports-2016-096175

20. Speers CJ, Bhogal GS. Greater trochanteric pain syndrome: a review of diagnosis and management in general practice. *Br J Gen Pract*. 2017;67(663):479-480. doi:10.3399/bjgp17X693041

21. Ostelo RWJG, by Vet HCW, Vlaeyen JWS, et al. Behavioral Graded Activity Following First-Time Lumbar Disc Surgery. *Spine (Phila Pa 1976)*. 2003;28(16):1757-1765. doi:10.1097/01.BRS.0000083317.62258.E6

22. Garcia SC, Dueweke JJ, Mendias CL. Optimal Joint Positions for Manual Isometric Muscle Testing. *J Sport Rehabil*. 2016;25(4). doi:10.1123/jsr.2015-0118

23. Picha KJ, Almaddah MR, Barker J, Ciochetty T, Black WS, Uhl TL. Elastic Resistance Effectiveness on Increasing Strength of Shoulders and Hips. *J Strength Cond Res*. 2019;33(4):931-943. doi:10.1519/JSC.0000000000002216

24. Zapparoli FY, Riberto M. Isokinetic Evaluation of the Hip Flexor and Extender Muscles: A Systematic Review. *J Sport Rehabil*. 2017;26(6):556-566. doi:10.1123/jsr.2016-0036

25. Sehn F, Chachamovich E, Vidor LP, et al. Cross-Cultural Adaptation and Validation of the Brazilian Portuguese Version of the Pain Catastrophizing Scale. *Pain Med*. 2012;13(11):1425-1435. doi:10.1111/j.1526-4637.2012.01492.x

26. Siqueira FB, Teixeira-Salmela LF, Magalhães L de C. Analysis of the psychometric properties of the Brazilian version of the kinesiophobia cap scale. *Orthopedic Act Bras*. 2007;15(1):19-24. doi:10.1590/S1413-78522007000100004

27. Caumo W, Antunes L, Lorenzzi Elkfury J, et al. The Central Sensitization Inventory validated and adapted for a Brazilian population: psychometric properties and its relationship with brain-derived neurotrophic factor. *J Pain Res*. 2017;Volume 10:2109-2122. doi:10.2147/JPR.S131479

28. Kraemer WJ, Adams K, Cafarelli E, et al. Progression models in resistance training for healthy adults. *Med Sci Sports Exercise*. 2002;34(2):364-380. doi:10.1097/00005768-200202000-00027

29. Tingley D, Yamamoto T, Hirose K, Keele L, Imai K. Mediation: R Package for Causal Mediation Analysis. *J Stat Software*. 2014;59(5). doi:10.18637/jss.v059.i05

30. Altman DG. The Revised CONSORT Statement for Reporting Randomized Trials: Explanation and Elaboration. *Ann Intern Med*. 2001;134(8):663. doi:10.7326/0003-4819-134-8-200104170-00012

31. Elkins MR, Moseley AM. Intention-to-treat analysis. *J Physiother*. 2015;61(3):165-167. doi:10.1016/j.jphys.205.05.013

32. Rio E, Kidgell D, Purdam C, et al. Isometric exercise induces analgesia and reduces inhibition in patellar tendinopathy. *Br J Sports Med*. 2015;49(19):1277-1283. doi:10.1136/bjsports-2014-094386

33.     Paiva E, Azevedo D, Pereira A , et al. Measurement properties of instruments used to assess patients with Greater Trochanteric Pain Syndrome (Under Review)

34. Costa RM de P, Cardinot TM, Mathias LNCDC, Leporace G, de Oliveira LP. Validation of the Brazilian version of the Hip Outcome Score (HOS) questionnaire. Adv Rheumatol [Internet]. 2018 Dec 24;58(1):4. Available from: https://advancesinrheumatology.biomedcentral.com/articles/10.1186/s42358-018-0007-y

35. Polesello GC, Godoy GF, Trindade CA de C, de Queiroz MC, Honda E, Ono NK. Translation and cross-cultural adaptation of the International Hip Outcome Tool (iHOT) into Portuguese. Ortop Bras. 2012;20(2):88-92B.
