# Supplementary material for: Motor control exercises versus general exercises for greater trochanteric pain syndrome: A protocol of a randomized controlled trial
Source: PLoS One. 2022 Jun 24;17(6):e0269230. doi: 10.1371/journal.pone.0269230 (PMC9231741; doi:10.1371/journal.pone.0269230)
Supplement: S3 Protocol — (PDF) [file pone.0269230.s004.pdf]

PARECER CONSUBSTANCIADO DO CEP

DADOS DA EMENDA

Título da Pesquisa: EXERCÍCIOS DE CONTROLE MOTOR VERSUS EXERCÍCIOS GERAIS NA SÍNDROME DOLOROSA DO TROCÂNTER MAIOR: ENSAIO CONTROLADO

Pesquisador: Guilherme Nava

Área Temática:

Versão: 4

CAAE: 87372318.1.0000.5406

Instituição Proponente: UNIVERSIDADE ESTADUAL PAULISTA JULIO DE MESQUITA FILHO

Patrocinador Principal: Financiamento Próprio

DADOS DO PARECER

Número do Parecer: 4.566.191

Apresentação do Projeto:

ntrodução: A síndrome dolorosa do trocânter maior (SDTM) é um termo abrangente usado para definir dor e sensibilidade na região do trocânter maior do fêmur, que é mais comum em mulheres. O controle anormal dos movimentos dos membros inferiores e parâmetros neuromusculares deficientes podem levar à SDTM, entretanto, nenhum estudo utilizou o treinamento neuromuscular como estratégia de tratamento. Objetivos: Assim, este estudo tem como objetivo comparar o efeito de um protocolo de exercícios gerais versus um programa de treinamento de controle motor sobre a dor no início e após o tratamento em mulheres com SDTM.

Métodos: Este estudo randomizado com 2 braços foi desenhado seguindo os itens do protocolo padrão para estudos de intervenção. Os resultados serão relatados de acordo com os padrões consolidados das diretrizes de relatórios de ensaios. Sessenta participantes serão randomizados para receber exercícios de controle motor ou exercícios gerais. As medidas de desfecho primário serão a pontuação da dor no quadril coletada no início do estudo, 8 semanas e 60 semanas.

Conclusão: Estudos têm sugerido que a SDTM pode estar relacionada ao controle pobre do quadril e da pélvis, entretanto, nenhum estudo investigou um protocolo de exercícios focado no aumento da força dos músculos abdutores e extensores do quadril associado ao treinamento do controle pélvico, especialmente em posições de suporte unilateral, como ocorre na marcha. Este estudo ajudará a determinar se a SDTM está relacionado ao controle anormal dos movimentos dos membros inferiores.

Objetivo da Pesquisa:

O objetivo deste estudo é comparar o efeito de um protocolo de exercícios gerais versus um programa de treinamento de controle motor sobre a dor no início e após o tratamento em 8 e 60 semanas em mulheres com SDTM.

Avaliação dos Riscos e Benefícios:

Não há riscos para os participantes do estudo. Os benefícios serão os resultados do estudo divulgado para a comunidade científica.

Comentários e Considerações sobre a Pesquisa:

A pesquisa está bem descrita e fundamentada teoricamente e o desenho dos estudo, da forma como está descrito permite observar que o mesmo será exequível conforme cronograma apresentado.

Considerações sobre os Termos de apresentação obrigatória:

Adequados.

Recomendações:

Não há.

Conclusões ou Pendências e Lista de Inadequações:

Não há pendências.

Considerações Finais a critério do CEP:

O CEP da FFC da UNESP de MARÍLIA, em reunião ordinária de 17/02/2021, após acatar o parecer do membro relator previamente aprovado para o presente estudo e atendendo a todos os dispositivos das resoluções 466/2012, 510/2016 e complementares, bem como ter aprovado o Termo de Consentimento Livre e Esclarecido como também todos os anexos incluídos na pesquisa, resolve APROVAR o projeto de pesquisa EXERCÍCIOS DE CONTROLE MOTOR VERSUS EXERCÍCIOS GERAIS NA SÍNDROME DOLOROSA DO TROCÂNTER MAIOR: ENSAIO CONTROLADO ALEATORIZADO

Este parecer foi elaborado baseado nos documentos abaixo relacionados:

Tipo Documento	Arquivo	Postagem	Autor	Situação
Declaração de Instituição e Infraestrutura	autorizacaocoleta.pdf	17/02/2021 12:25:55	CLAUDIO ROBERTO BROCANELLI	Aceito
Cronograma	cronogramaatualizado.docx	17/02/2021	CLAUDIO ROBERTO	Aceito

Endereço: Av. Hygino Muzzi Filho, 737
Bairro: Campus Universitário **CEP:** 17.525-900
UF: SP **Município:** MARILIA
Telefone: (14)3402-1346 **E-mail:** cep.marilia@unesp.br

Cronograma	cronogramaatualizado.docx	12:25:38	BROCANELLI	Aceito
Informações Básicas do Projeto	PB_INFORMAÇÕES_BÁSICAS_1664577_E2.pdf	18/01/2021 16:11:49		Aceito
Projeto Detalhado / Brochura Investigador	Projeto_doutorado_CEP.docx	18/01/2021 16:02:51	Guilherme Nava	Aceito
Outros	Curriculo_Marcelo_Navega.pdf	16/11/2020 21:33:28	Guilherme Nava	Aceito
Outros	Curriculo_Guilherme_Nava.pdf	16/11/2020 21:32:53	Guilherme Nava	Aceito
Declaração de Instituição e Infraestrutura	Autorizacao_da_instituicao.pdf	16/11/2020 21:31:29	Guilherme Nava	Aceito
TCLE / Termos de Assentimento / Justificativa de Ausência	TCLE.docx	16/11/2020 21:29:35	Guilherme Nava	Aceito
Folha de Rosto	Folha_de_rosto.pdf	16/11/2020 21:28:16	Guilherme Nava	Aceito

Situação do Parecer:
Aprovado

Necessita Apreciação da CONEP:
Não

MARILIA, 01 de Março
de 2021

Assinado por:
SIMONE APARECIDA CAPELLINI
(Coordenador(a))

Endereço: Av. Hygino Muzzi Filho, 737
Bairro: Campus Universitário **CEP:** 17.525-900
UF: SP **Município:** MARILIA
Telefone: (14)3402-1346 **E-mail:** cep.marilia@unesp.br
