# Supplementary material for: Motor control exercises versus general exercises for greater trochanteric pain syndrome: A protocol of a randomized controlled trial
Source: PLoS One. 2022 Jun 24;17(6):e0269230. doi: 10.1371/journal.pone.0269230 (PMC9231741; doi:10.1371/journal.pone.0269230)
Supplement: Attachment — Submitted filename: Response to Reviewers.docx [file pone.0269230.s005.docx]

Reviewer #1

It is advisable not to use abbreviations in the abstract.

Thank you for the guidance, the change has been made.

Line 146- when they talk "the second one will be carried out by a blinded-assessor", it is not clear what this assessor is doing in this sentence. In fact it is not clear from the manuscript what this evaluator does. Please clarify what tasks this evaluator does in the manuscript, as it is not clear, nor how many times he/she evaluates the subject (line 211).

Thank you for the guidance, the change has been made in the manuscript. “The second assessment will be carried out by two different blinded assessors, both registered physiotherapists with PhD in musculoskeletal disorders, to evaluate the participants outcomes. They are pain intensity, global perceived effect, muscle strength, pain catastrophization, kinesiophobia, central sensitization and quality of life. The blinded assessors will evaluate the participants at baseline (before the treatment), after 8 weeks of intervention and after 60 weeks of intervention.”

At what point do the participants sign the informed consent, according to the Randomization, allocation and blinding section, is when they are going to receive the intervention program. The informed consent should be signed at the moment that the subjects want to participate once they have been thoroughly explained what the study consists of.

Thank you for the guidance, the change has been made.

“Randomization, allocation and blinding: If all eligibility criteria are fulfilled, and if the presence of GTPS is determined, the participants will be randomly assigned to receive either 1) motor control exercises or 2) general exercises (fig 2). At this point, before the blind evaluation, participants will give their free and informed consent.”

Review bibliography number 28, it does not follow the format.

We revised the bibliography using mendeley software. We believe that the error in the bibliographic references has been corrected.

Reviewer #2

Line 197. “effect size of 0,5” Please clarify whether this is in standard deviation unit? If not, what standard deviation was used in this power analysis.

Thank you for the guidance, the change has been made.

“The sample size calculation was performed through the G * Power software, and was based on the ability to detect a difference of 2 points (pilot standard deviation of ±0,8 points) of pain intensity score, because it is the primary outcome of the research. A power of 0.80, probability of error α 0.05, effect size of 0,48 and a 15% dropout rate were used, thus we will recruit 60 participants (30 in each group)”

Line 370. “appropriate statistical analyses will be adopted for the variables”. Please be specific for “appropriate statistical analyses”.

Thank you for the guidance, the change has been made.

Line 377. “A causal mediation analysis will be conducted..” please be specific what mediation analysis will be conducted.

Thank you for the guidance, the change has been made.

Reviewer #3

I am pleased to see the VISA-G outcome tool is mentioned within this protocol to improve homogenous outcome measures within GTPS research. However, it is unclear why this is not included as an outcome within the protocol submitted after ethical approval of the original document. It is unclear if the VISA-G is to be used as an outcome measure or not. If it is, please add this into either the primary or secondary outcomes. If it is not to be used as an outcome measure, why not? This has been previously recommended to be used in GTPS research to improve outcome homogeneity.

We appreciate the question and the opportunity to answer it. We understand the importance of using this questionnaire to assess the disability outcome. When we started the study, we discovered that the VISA-G questionnaire would be translated, culturally adapted, and validated for the population with greater trochanter pain syndrome, however, we had to start data collection before the publication of the article validating the VISA-G for the Brazilian Portuguese language, which only took place in early 2021.

Therefore, of the aforementioned questionnaires, we only used the International hip outcome tool. Unfortunately, we realized the error only after submission, we forgot to remove the information from the "ethical aspects", as well as, to add the assessment of the quality of life in the secondary outcomes.

You have stated that greater tochanteric palpation in conjunction with one other test will be used. It would be informative to state that this is because GT palp is of little benefit when the test is positive due to the poor Sp. Therefore, in the absence of a negative GT palp test, another test with high Sp will be used to rule in the pathology of interest (GTPS) to reduce your false positive inclusion rate.

Thank you for the guidance, the change has been made.

“The presence of pain on palpation is an inclusion criteria, therefore, it is mandatory for participation in the study.

- Palpation of the greater trochanter: positive palpation tenderness, considered when there is pain over the greater trochanter of the femur, during the evaluation in lateral decubitus (LD) with the pain side up, flexion of the hip joint and knees together[5,6,8,11,18].

Because of the little benefit of the greater trochanteric palpation when the test is positive, due to the poor specificity, another test with high specificity will be used to rule in the greater trochanteric pain syndrome, to reduce false positive. Additionally to the positive greater trochanteric palpation test, the participants also must experience pain in one or more of the six provocative clinical pain tests[13,17,18]. They are:”

Changes to the reference list

Due to the changes suggested by the reviewers, we have added 2 new references:

Reference 29, due to the addition of a study outcome.

Reference 31, due to changes made in the statistical analysis.
